# Supplementary material for: Causal Relationship between Aging and Anorexia Nervosa: A White-Matter-Microstructure-Mediated Mendelian Randomization Analysis
Source: Biomedicines. 2024 Aug 16;12(8):1874. doi: 10.3390/biomedicines12081874 (PMC11351342; doi:10.3390/biomedicines12081874)
Supplement: Supplementary file 1 [file biomedicines-12-01874-s001.zip › Supplementary Figure/Figure S4/Singe SNP plot of MR analysis for TL to ubm-b-1509.pdf]

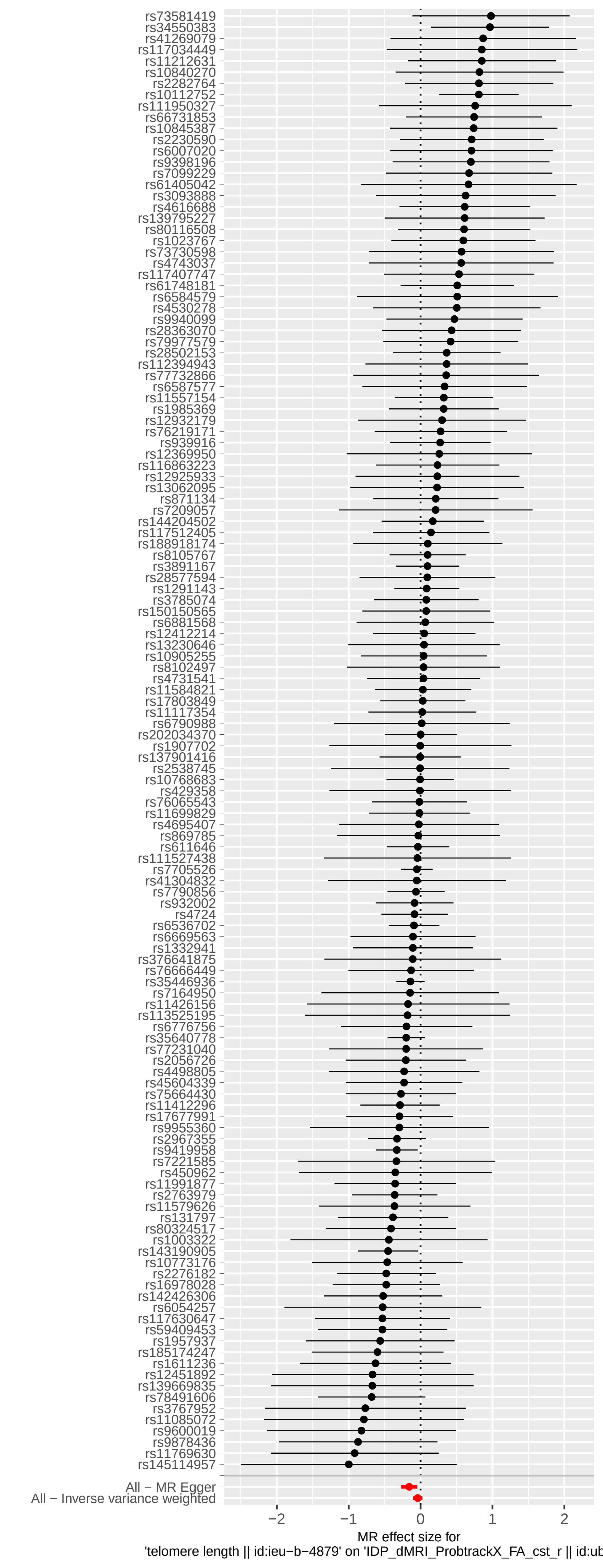
rs73581419
rs34550383
rs41269079
rs117034449
rs11212631
rs10840270
rs2282764
rs10112752
rs111950327
rs66731853
rs10845387
rs2230590
rs6007020
rs9398196
rs7099229
rs61405042
rs3093888
rs4616688
rs139795227
rs80116508
rs1023767
rs73730598
rs4743037
rs117407747
rs61748181
rs6584579
rs4530278
rs9940099
rs28363070
rs79977579
rs28502153
rs112394943
rs77732866
rs6587577
rs11557154
rs1985369
rs12932179
rs76219171
rs939916
rs12369950
rs116863223
rs12925933
rs13062095
rs871134
rs7209057
rs144204502
rs117512405
rs188918174
rs8105767
rs3891167
rs28577594
rs1291143
rs3785074
rs150150565
rs6881568
rs12412214
rs13230646
rs10905255
rs8102497
rs4731541
rs11584821
rs17803849
rs11117354
rs6790988
rs202034370
rs1907702
rs137901416
rs2538745
rs10768683
rs429358
rs76065543
rs11699829
rs4695407
rs869785
rs611646
rs111527438
rs7705526
rs41304832
rs7790856
rs932002
rs4724
rs6536702
rs6669563
rs1332941
rs376641875
rs76666449
rs35446936
rs7164950
rs11426156
rs113525195
rs6776756
rs35640778
rs77231040
rs2056726
rs4498805
rs45604339
rs75664430
rs11412296
rs17677991
rs9955360
rs2967355
rs9419958
rs7221585
rs450962
rs11991877
rs2763979
rs11579626
rs131797
rs80324517
rs1003322
rs143190905
rs10773176
rs2276182
rs16978028
rs142426306
rs6054257
rs117630647
rs59409453
rs1957937
rs185174247
rs1611236
rs12451892
rs139669835
rs78491606
rs3767952
rs11085072
rs9600019
rs9878436
rs11769630
rs145114957
All − MR Egger
All − Inverse variance weighted
−2
−1
0
1
2
MR effect size for
'telomere length || id:ieu−b−4879' on 'IDP_dMRI_ProbtrackX_FA_cst_r || id:ub
